# Parasite-Specific CD4^+^ IFN-γ^+^ IL-10^+^ T Cells Distribute within Both Lymphoid and Nonlymphoid Compartments and Are Controlled Systemically by Interleukin-27 and ICOS during Blood-Stage Malaria Infection

**DOI:** 10.1128/IAI.01100-15

**Published:** 2015-12-28

**Authors:** Ana Villegas-Mendez, Tovah N. Shaw, Colette A. Inkson, Patrick Strangward, J. Brian de Souza, Kevin N. Couper

**Affiliations:** a Faculty of Life Sciences, University of Manchester, Manchester, United Kingdom; b Institute of Immunity and Transplantation, UCL Department of Immunology, Royal Free Hospital, London, United Kingdom

## Abstract

Immune-mediated pathology in interleukin-10 (IL-10)-deficient mice during blood-stage malaria infection typically manifests in nonlymphoid organs, such as the liver and lung. Thus, it is critical to define the cellular sources of IL-10 in these sensitive nonlymphoid compartments during infection. Moreover, it is important to determine if IL-10 production is controlled through conserved or disparate molecular programs in distinct anatomical locations during malaria infection, as this may enable spatiotemporal tuning of the regulatory immune response. In this study, using dual gamma interferon (IFN-γ)–yellow fluorescent protein (YFP) and IL-10–green fluorescent protein (GFP) reporter mice, we show that CD4^+^ YFP^+^ T cells are the major source of IL-10 in both lymphoid and nonlymphoid compartments throughout the course of blood-stage Plasmodium yoelii infection. Mature splenic CD4^+^ YFP^+^ GFP^+^ T cells, which preferentially expressed high levels of CCR5, were capable of migrating to and seeding the nonlymphoid tissues, indicating that the systemically distributed host-protective cells have a common developmental history. Despite exhibiting comparable phenotypes, CD4^+^ YFP^+^ GFP^+^ T cells from the liver and lung produced significantly larger quantities of IL-10 than their splenic counterparts, showing that the CD4^+^ YFP^+^ GFP^+^ T cells exert graded functions in distinct tissue locations during infection. Unexpectedly, given the unique environmental conditions within discrete nonlymphoid and lymphoid organs, we show that IL-10 production by CD4^+^ YFP^+^ T cells is controlled systemically during malaria infection through IL-27 receptor signaling that is supported after CD4^+^ T cell priming by ICOS signaling. The results in this study substantially improve our understanding of the systemic IL-10 response to malaria infection, particularly within sensitive nonlymphoid organs.

## INTRODUCTION

The control and resolution of blood-stage malaria infection are mediated through dynamic and bidirectional interactions between effector and regulatory components of the immune system. Thus, the generation of excessive proinflammatory innate and/or adaptive immune responses due to failed regulation invariably leads to the formation of fulminant immunopathology, even if parasite killing is extremely effective (1–4). Conversely, the failure to mount adequate antiparasitic immune responses in the face of mistimed or overly strong regulatory responses enables parasite outgrowth, hyperparasitemia, and associated complications (1–4).

The regulatory cytokine interleukin-10 (IL-10) plays a critical role in controlling the outcome of blood-stage murine malaria infection: IL-10-deficient mice generally exhibit lower parasite burdens than their wild-type counterparts during blood-stage malaria infection, indicating that antiparasitic immune responses are potentiated in the absence of IL-10 (5–8). However, IL-10-deficient mice exhibit increased inflammatory processes and frequently develop severe immune-mediated pathology during various Plasmodium species infections (5–8). Moreover, IL-10 contributes to the protection against experimental cerebral malaria observed in mice with helminth or heterologous malaria parasite coinfections, as well as that induced in mice following repeated rounds of infection and drug cure (9–11). IL-10 also significantly influences the course of human malaria infection, with genetic polymorphisms in the IL-10 gene being associated with protection or susceptibility to infection (12, 13). More generally, the ratio of IL-10 to proinflammatory mediators, such as tumor necrosis factor (TNF), appears to determine the effectiveness of parasite clearance and the development of symptomatic or severe malarial disease (12, 14–18). Notably, the host-protective roles of IL-10 have also been demonstrated in many other infections and autoimmune conditions (1, 4, 19, 20), establishing IL-10 as an instrumental component of the immune regulatory network operational during inflammation.

Immune-mediated pathology typically manifests in nonlymphoid organs, such as the liver, lung, and brain, in IL-10-deficient mice during malaria infection (5, 7, 21). This suggests that IL-10 plays a key regulatory role within these tissue sites in regulating tissue-damaging inflammation during infection. However, to date, the cellular source of IL-10 during blood-stage malaria infection has been examined only in the spleen in mice (5, 22, 23) and in the blood of humans (24, 25). Consequently, the cellular source of IL-10 in the sensitive nonlymphoid organs is unknown. As such, we have a very limited comprehension of the overall systemic protective IL-10 response during blood-stage malaria infection. Notably, although CD4^+^ T cells appear to be the predominant source of IL-10 in the spleen in mice (5, 22, 23) and blood in humans (24, 25), during blood-stage malaria infection, IL-10 can be produced by virtually all leukocyte populations (19).

It is also clear that distinct nonlymphoid tissue sites, such as the liver and lung, present environmental conditions different from those in the spleen at homeostasis and during inflammation (26, 27). Thus, although IL-27 appears to instruct IL-10 production by splenic Th1 cells during Plasmodium chabaudi AS infection and during a number of other infections (22, 28), it is unknown if conserved or distinct molecular pathways program IL-10 production by leukocytes in different anatomical locations during blood-stage malaria infection or any infection. Of relevance, a myriad of context-dependent pathways can instruct and/or stabilize IL-10 expression by CD4^+^ T cell subsets (1, 29).

In this study, we have performed a spatiotemporal investigation of the host-protective IL-10 response during blood-stage P. yoelii infection, to address whether tissue-specific environmental signals variably control the source and magnitude of the IL-10 response in distinct anatomical locations. We show using dual gamma interferon (IFN-γ)–yellow fluorescent protein (YFP) and IL-10–green fluorescent protein (GFP) reporter mice that antigen-experienced CD4^+^ IFN-γ^+^ T cells are the dominant source of IL-10 in both lymphoid and nonlymphoid compartments during the course of P. yoelii infection. Adoptively transferred mature splenic CD4^+^ YFP^+^ GFP^+^ T cells were able to migrate to and accumulate within all examined organs of malaria parasite-infected mice, yet CD4^+^ YFP^+^ GFP^+^ T cells exhibited differences in their functional characteristics in the liver and lung from those in the spleen. Finally, we show that the programming and/or maintenance of IL-10 expression by CD4^+^ IFN-γ^+^ T cells is controlled systemically by IL-27 receptor (IL-27R) signaling, which requires subsequent ICOS signaling postpriming. Combined, our results provide new information on the spatial distribution and pathways controlling CD4^+^ YFP^+^ GFP^+^ T cell development and maintenance during malaria infection.

## MATERIALS AND METHODS

### Ethics.

All animal work was approved following local ethical review by the University of Manchester Animal Procedures and Ethics Committee and was performed in strict accordance with the United Kingdom Home Office Animals (Scientific Procedures) Act 1986 (project license 70/7293).

### Mice and parasites.

C57BL/6 CD45.1^+^ mice (Pep3), IFN-γ–YFP reporter mice (30), and IL-10–GFP reporter mice (31) were bred and maintained at the University of Manchester in individual ventilated cages. Dual-reporter IFN-γ and IL-10 mice were generated by intercrossing heterozygous IFN-γ reporter mice with homozygous IL-10 reporter mice. F1 offspring were used in experiments after genotyping for YFP expression by flow cytometry. Sex-matched 6- to 10-week-old mice were used in separate experiments.

Cryopreserved P. yoelii NL parasites were thawed and passaged once through C57BL/6 mice before being used to infect experimental mice. Mice were infected with 1 × 10^4^ parasitized red blood cells (pRBCs) via intravenous (i.v.) injection in the tail vein. In some experiments, experimental mice were injected intraperitoneally (i.p.), separately, with 250 μg anti-WSX-1, 250 μg anti-IL-12p40 (clone C17.8), 250 μg anti-ICOS (clone 17G9), 250 μg anti-transforming growth factor β (anti-TGF-β; clone 1D11.16.8), 250 μg anti-IL-2 (clone JES6-5H4), and 250 μg anti-CD70 (clone FR-70) monoclonal antibodies (MAbs) on days −1, +1, +3, and +5 of infection. In some experiments, anti-ICOS MAbs were administered from day 3 of infection. All antibodies were from BioXcell (West Lebanon, NH), except for the anti-WSX-1 MAb, which was obtained from Amgen, Inc. (Seattle, WA). The course of infection was monitored every second day of infection (from day 3 postinfection [p.i.]) by microscopic examination of peripheral parasite levels in Giemsa-stained thin blood smears.

### Flow cytometry.

Spleens, livers, and Peyer's patches were collected from naive and malaria parasite-infected mice, and single-cell suspensions were prepared by homogenization through a 70-μm-mesh-size cell strainer (BD Biosciences). Red blood cells (RBCs) were lysed (RBC lysing buffer; BD Biosciences), and samples were washed and resuspended in fluorescence-activated cell sorting (FACS) buffer (Hanks balanced salt solution [HBSS] with 2% fetal calf serum [FCS]). Blood was treated with RBC lysis buffer and resuspended in FACS buffer. Lungs were roughly dissected into small chunks, aspirated through a 10-ml syringe, and incubated in HBSS containing 2% FCS with collagenase (final concentration, 1 mg/ml; Sigma) for 45 min on a tube roller at room temperature. The resulting suspension was filtered through a 70-μm-mesh-size cell strainer, RBCs were lysed, and samples were refiltered through a 70-μm-mesh size cell strainer. The samples were then washed and resuspended in FACS buffer. Live/dead cell differentiation and absolute cell numbers were calculated by trypan blue exclusion (Sigma-Aldrich) using a C-Chip hemocytometer (NanoEnTek, Pleasanton, CA).

All FACS samples were first stained with LIVE/DEAD fixable blue dead cell stain for UV (Life Technologies). CD4^+^ T cell phenotyping was performed by surface staining with anti-mouse antibodies (clones indicated in parentheses) against CD4 (GK1.5), CD44 (IM7), CD62L (MEL-14), CD49d (R1-2), CD11a (M17/4), CCR1 (643854), CCR5 (HM-CCR5 7A4), CXCR6 (221002), CCR6 (29-2L17), CXCR3 (CXCR3-173), KLRG1 (2F1), PD-1 (RMP1-30), CD25 (PC61), LAG-3 (C9B7W), CD69 (H1.2F3), ICOS (C398.4A), Tigit (GIGD7), and CD103 (2E7). Different lymphoid populations were assessed by surface staining with CD3 (17A2), CD8a (53-6.7), CD11c (N418), CD49b (DX5), F4-80 (BM8), Ly6C (HK1.4), CD19 (6D5), and CD11b (M1/70). All staining cocktails contained the mouse Fc-receptor block (clone Fc-G2a).

All antibodies were purchased from eBioscience, BioLegend, or R&D Systems. Fluorescence-minus-one (FMO) controls were used to validate flow cytometric results. All flow cytometry acquisition was performed using an LSR II flow cytometer (BD Systems, United Kingdom) under the same application settings, with a configuration including 510/20 band-pass (BP) and 500 long-pass (LP) filters (for the GFP signal) and 550/30 BP and 525 LP filters (for the YFP signal). All FACS analyses were performed using FlowJo software (TreeStar Inc., OR, USA).

### Adoptive transfer experiments.

Splenic CD4^+^ T cells from malaria parasite-infected IFN-γ and IL-10 dual-reporter mice were positively selected by magnetic cell sorting using anti-CD4 microbeads (Miltenyi Biotech) according to the manufacturer's guidelines. Purified CD4^+^ T cells were then stained with anti-mouse CD49d and anti-mouse CD11a (as described above), and CD4^+^ CD49d^+^ CD11a^+^ YFP^+^ GFP^−^ and CD4^+^ CD49d^+^ CD11a^+^ YFP^+^ GFP^+^ T cells were obtained by flow cytometric cell sorting (BD Aria). The cells were then adoptively transferred by i.v. injection into naive or malaria parasite-infected (day 7 of infection) congenic CD45.1^+^ mice.

### Quantification of cytokine secretion by CD4^+^ T cells.

CD4^+^ CD49d^+^ CD11a^+^ YFP^+^ GFP^−^ and CD4^+^ CD49d^+^ CD11a^+^ YFP^+^ GFP^+^ T cells were sort purified, as described above, from the spleens, livers, and lungs of infected (day 7 of infection) dual-reporter IFN-γ and IL-10 mice and cultured overnight at a density of 1 × 10^5^ cells on 96-well plates with or without 2 μg/ml anti-CD3 (BD Biosciences) and 2 μg/ml anti-CD28 (eBioscience). Cell supernatants were stored at −80°C until further use. The concentrations of IL-2, IL-4, IL-6, IFN-γ, TNF, IL-17A, and IL-10 in the supernatants were measured by a cytometric bead array (CBA) mouse Th1/Th2/Th17 cytokine kit (BD Biosciences), following the manufacturer's instructions.

### Statistical analysis.

Data were first assessed using the D'Agostino and Pearson omnibus normality test. For comparisons of two groups, statistical significance was determined using the *t* test or the Mann-Whitney U test. For comparisons of three or more groups, statistical significance was determined using a one-way or two-way analysis of variance (ANOVA) with Tukey *post hoc* analysis or a Kruskal-Wallis test with Dunn *post hoc* analysis. Results were considered significantly different when *P* was <0.05.

## RESULTS

### CD4^+^ T cells are the major source of IL-10 in both lymphoid and nonlymphoid compartments during malaria infection.

**FIG 1:**
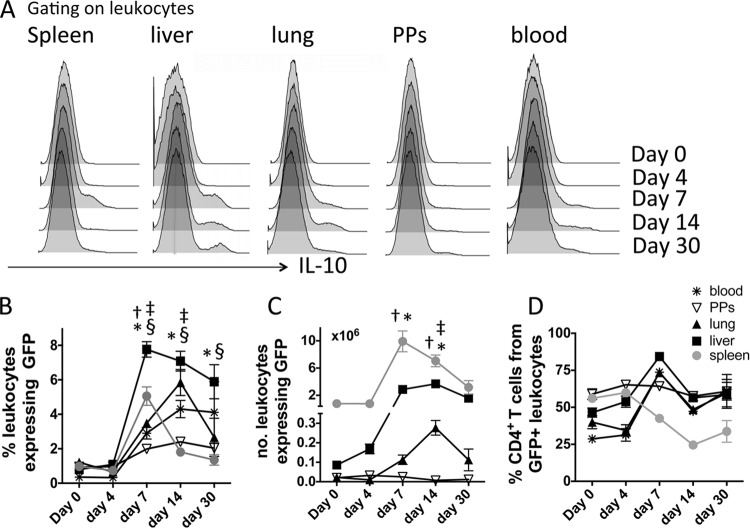
The kinetics of IL-10 production in different lymphoid and nonlymphoid locations during the course of blood-stage P. yoelii NL infection. IFN-γ and IL-10 dual-reporter mice were infected (i.v.) with 1 × 10^4^ P. yoelii NL pRBCs. (A) Representative histograms showing the frequencies of total IL-10–GFP^+^ events in different tissues throughout the course of infection. (B, C) The calculated frequencies (B) and absolute numbers (C) of IL-10–GFP^+^ cells in the tissues throughout the course of infection. (D) The calculated contribution of CD4^+^ T cells to the overall tissue IL-10–GFP responses throughout the course of infection. (B to D) The results are the mean ± SEM for the group from 4 to 5 independent experiments (3 to 5 mice in each experiment). †, *P* < 0.05 between the annotated day of infection and day 0 (spleen); *, *P* < 0.05 between the annotated day of infection and day 0 (liver); ‡, *P* < 0.05 between the annotated day of infection and day 0 (lung); §, *P* < 0.05 between the annotated day of infection and day 0 (blood). Significance was tested using a two-way ANOVA with Tukey *post hoc* analysis. PPs, Peyer's patches.

To address the question of whether CD4^+^ T cells were the major source of IL-10 in sensitive nonlymphoid compartments as well as the spleen throughout the course of blood-stage malaria infection, we infected IFN-γ–YFP and IL-10–GFP dual-reporter mice and first quantified the total IL-10 response in various tissue sites. The frequencies of IL-10–GFP^+^ cells increased to variable levels in all examined tissues by day 7 of infection (Fig. 1A and B). The kinetics of the IL-10–GFP response were, however, heterogeneous in the different tissue sites over the course of infection; the numbers of IL-10-producing cells declined rapidly from a peak on day 7 of infection in the spleen, whereas the expansion and contraction were more gradual in the other organs, with the exception of the Peyer's patches, where there was no significant increase in the numbers of IL-10–GFP^+^ events throughout the course of infection (Fig. 1C). CD4^+^ T cells constituted the dominant source of IL-10 (typically >50% of the total IL-10 response) in all examined organs throughout the course of infection, with the notable exceptions of the spleen (day 14 p.i. or later) and the blood (day 4 p.i.) (Fig. 1D). Overall, the contribution of CD4^+^ T cells to the total IL-10 response was lowest in the spleen, indicating that other cells (which include B cells, dendritic cells/macrophages, and NK cells [data not shown]) also produce IL-10 in this site during the course of malaria infection. The contribution of CD4^+^ T cells to the total IL-10 response in the spleen was likely lower than that which we have previously reported (5), as the Vert-X mice utilized in this study more sensitively report IL-10 expression from macrophages and innate cells than the Tiger mice utilized previously (31).

### Antigen-experienced IFN-γ-producing CD4^+^ T cells are the dominant source of IL-10 in nonlymphoid and lymphoid compartments during blood-stage P. yoelii infection.

**FIG 2:**
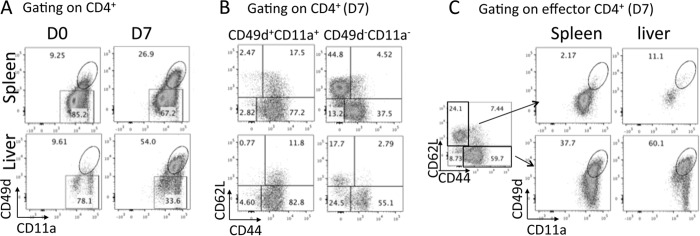
CD49d and CD11a coexpression define infection-induced, antigen-experienced effector CD4^+^ T cell populations. (A) Representative dot plots showing the expression of CD11a and CD49d on splenic and hepatic CD4^+^ T cells in naive and infected (P. yoelii NL, day 7 p.i.) mice; (B) representative dot plots showing the expression of CD44 and CD62L on CD4^+^ CD11a^−^ CD49d^−^ and CD4^+^ CD11a^+^ CD49d^+^ T cell subsets from the spleen and liver of infected (P. yoelii NL, day 7 p.i.) mice; (C) representative dot plots showing the expression of CD49d and CD11a on naive CD4^+^ CD44^−^ CD62L^hi^ T cells and activated CD4^+^ CD44^+^ CD62L^low^ T cells from the spleen and liver of infected (P. yoelii NL, day 7 p.i.) mice. D0 and D7, days 0 and 7 p.i., respectively.

CD4^+^ T cells were a major source of IL-10 in all examined organs during malaria infection, with the exception of the spleen after day 14 of infection. Thus, we next addressed whether the cytokine was produced by equivalent (or different) helper T cell subsets in the diverse tissue environments during infection. To do this, we first adopted the gating strategy developed by Butler et al. (32), utilizing coexpression of CD49d and CD11a to identify antigen-experienced effector CD4^+^ T cells, which include all or the majority of recently activated parasite-specific CD4^+^ T cells. This surrogate marker system was necessary, as ovalbumin (OVA)-expressing P. yoelii NL parasites have not been generated and major histocompatibility complex class II (I-A^b^) tetramers for P. yoelii do not exist. As expected, very few CD4^+^ CD11a^+^ CD49d^+^ T cells were observed in naive mice, and this population dramatically expanded systemically, with the exception of in Peyer's patches, during malaria infection (Fig. 2A and results not shown). The CD4^+^ CD11a^+^ CD49d^+^ T cells were uniformly CD44^+^ and predominantly CD62L^−^ in all tissue sites on all days examined (Fig. 2B and results not shown). Conversely, a significant frequency of CD44^+^ CD62L^−^ cells, particularly in the spleen, did not express CD49d and CD11a during infection (Fig. 2C).

**FIG 3:**
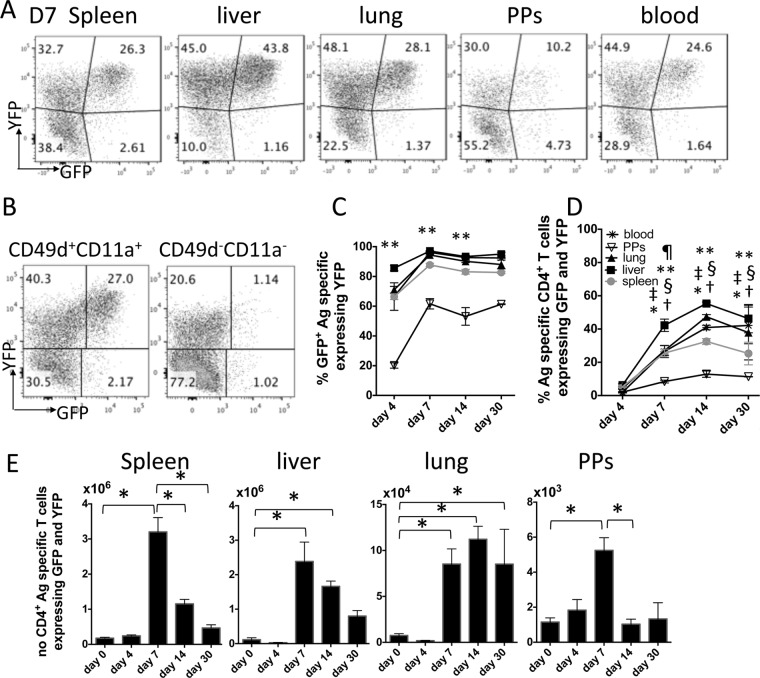
Antigen-experienced CD4^+^ IFN-γ–YFP^+^ T cells are the dominant source of IL-10 in lymphoid and nonlymphoid tissues during malaria infection. IFN-γ and IL-10 dual-reporter mice were infected (i.v.) with 1 × 10^4^ P. yoelii NL pRBCs. (A) Representative histograms showing the expression of IFN-γ–YFP and IL-10–GFP by antigen-experienced (CD11a^+^ CD49d^+^) CD4^+^ T cells in the different tissues on day 7 of infection. (B) Representative dot plots showing the expression of IFN-γ–YFP and IL-10–GFP by gated splenic CD4^+^ CD11a^+^ CD49d^+^ and CD4^+^ CD11a^−^ CD49d^−^ T cells on day 7 of infection. (C) The proportion of IL-10–GFP^+^ antigen (Ag)-experienced CD4^+^ T cells that coexpressed IFN-γ–YFP in the different tissues over the course of infection. (D) The frequencies of parasite-specific CD4^+^ T cells in the different tissues that coexpressed IFN-γ–YFP and IL-10–GFP during infection. (E) The calculated numbers of parasite-specific CD4^+^ IFN-γ–YFP^+^ IL-10–GFP^+^ cells in the different tissues during the course of infection. The results are the mean ± SEM for the group from 4 to 5 independent experiments (3 to 5 mice in each experiment). (C, D) **, *P* < 0.05 between Peyer's patches and all other organs on the annotated day of infection; ¶, *P* < 0.05 between the liver and all other organs on the annotated day of infection; †, *P* < 0.05 between the annotated day of infection and day 4 (spleen); *, *P* < 0.05 between the annotated day of infection and day 4 (liver); ‡, *P* < 0.05 between the annotated day of infection and day 4 (lung); §, *P* < 0.05 between the annotated day of infection and day 4 (blood). Significance was tested using two-way ANOVA with Tukey *post hoc* analysis. (E) *, *P* < 0.05. Significance was tested using one-way ANOVA with Tukey *post hoc* analysis.

Gating onto CD4^+^ CD11a^+^ CD49d^+^ T cells in the IFN-γ–YFP and IL-10–GFP dual-reporter mice identified that YFP^+^ cells were the dominant (almost exclusive) source of IL-10 in all anatomical compartments throughout the course of malaria infection (Fig. 3A to C). The notable exception was the Peyer's patches, where an average of 50% of IL-10^+^ T cells were YFP^−^ (Fig. 3C). Of note, in all locations, YFP^+^ GFP^+^ cells were found only in the CD4^+^ CD11a^+^ CD49d^+^ T cell subset, whereas YFP^+^ GFP^−^ cells were also found in the CD4^+^ CD11a^−^ CD49d^−^ (but not CD44^+^ CD62L^−^) pool (Fig. 3B). The frequencies of CD4^+^ CD11a^+^ CD49d^+^ T cells expressing YFP and GFP increased rapidly from day 4 in all tissues, with the exception of the Peyer's patches, largely stabilizing in the spleen by day 7 p.i. and in the other organs by day 14 p.i. (Fig. 3D). The numbers of CD4^+^ CD11a^+^ CD49d^+^ YFP^+^ GFP^+^ T cells transiently peaked on day 7 in the spleen, liver, and Peyer's patches, whereas the numbers were maintained (at day 7 levels) in the lung (Fig. 3E). Thus, IL-10 is expressed primarily by antigen-experienced, putatively parasite-specific, CD4^+^ IFN-γ^+^ T cells in all examined lymphoid and nonlymphoid tissue sites, with the exception of gut-associated lymphoid organs, during malaria infection.

**FIG 4:**
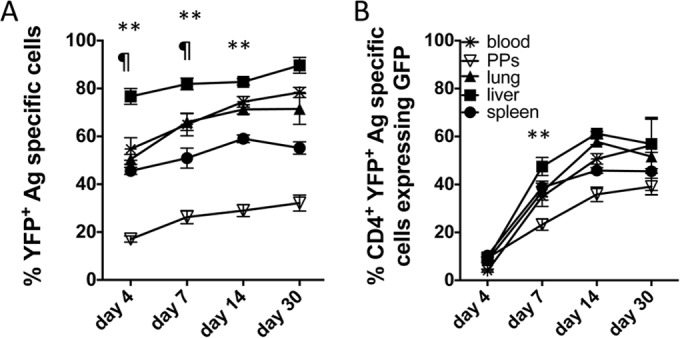
A conserved tissue-independent mechanism equilibrates the ratio of antigen-experienced CD4^+^ IFN-γ–YFP^+^ and CD4^+^ IFN-γ–YFP^+^ IL-10–GFP^+^ populations during malaria infection. IFN-γ and IL-10 dual-reporter mice were infected (i.v.) with 1 × 10^4^ P. yoelii NL pRBCs. (A) The frequencies of antigen-experienced CD4^+^ T cells in the different tissues that express IFN-γ–YFP during the course of infection. (B) The proportion of IFN-γ–YFP^+^ parasite-specific CD4^+^ T cells that coexpressed IL-10–GFP in the different tissues over the course of infection. The results are the mean ± SEM for the group from 4 to 5 independent experiments (3 to 5 mice in each experiment). **, *P* < 0.05 between the Peyer's patches group and all other groups (at the corresponding time point); ¶, *P* < 0.05 between the liver group and all other groups (at the corresponding time point). Significance was tested using two-way ANOVA with Tukey *post hoc* analysis.

The plateau in the magnitude of the IL-10 response was variable between tissues, with significantly higher frequencies of YFP^+^ GFP^+^ cells (out of the CD4^+^ CD11a^+^ CD49d^+^ population) being observed in the liver than in the spleen (Fig. 3D). We therefore questioned whether this was due to enrichment of antigen-experienced CD4^+^ YFP^+^ GFP^+^ T cells at the expense of CD4^+^ YFP^+^ GFP^−^ T cells in these tissue sites. Gating on total antigen-experienced CD4^+^ CD11a^+^ CD49d^+^ YFP^+^ T cells, we observed increased frequencies of total YFP^+^ cells within the antigen-experienced CD4^+^ T cell population in the liver compared with their frequencies in the other tissues (Fig. 4A). Very few antigen-experienced CD4^+^ T cells expressed YFP in the Peyer's patches, further indicating that blood-stage malaria infection does not provoke a significant intestinal CD4^+^ T cell response. Interestingly, however, the balance between GFP^+^ and GFP^−^ cells within the (disparately sized) CD4^+^ YFP^+^ T cell populations achieved parity in all examined tissues by day 7 of infection (Fig. 4B). Indeed, the percentage of YFP^+^ T cells coexpressing IL-10–GFP appeared to reach an upper, largely consistent threshold of 50 to 60% in all tissue sites examined (Fig. 4B). This suggests that, irrespective of tissue location, the level of IL-10 production by the Th1 cell population may be tightly calibrated during infection.

### Splenic CD4^+^ IFN-γ^+^ IL-10^+^ T cells can migrate to and enter nonlymphoid tissues during blood-stage malaria infection.

**FIG 5:**
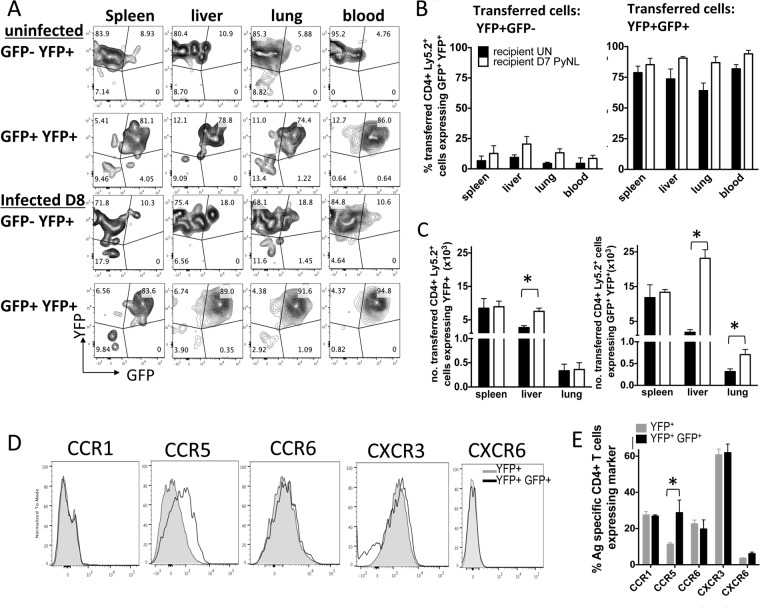
Mature splenic IFN-γ^+^ IL-10^+^ CD4^+^ T cells can migrate to and accumulate in nonlymphoid tissues during malaria infection. (A to E) Splenic antigen-experienced (CD11a^+^ CD49d^+^) IFN-γ–YFP^+^ GFP^+/−^ CD4^+^ T cells were sort purified from P. yoelii NL-infected (day 7, 1 × 10^4^ pRBCs [i.v.]) dual-reporter mice and were adoptively transferred separately into infected (day 7 p.i.) or naive congenic CD45.1^+^ mice. (A) Representative dot plots showing the expression of IFN-γ–YFP and IL-10–GFP by donor (CD45.2^+^) cells in the spleen, liver, lung, and blood of naive and infected recipients 1 day posttransfer. (B) The frequencies of transferred CD4^+^ YFP^+^ GFP^−^ cells (left) and CD4^+^ YFP^+^ GFP^+^ cells (right) expressing IL-10–GFP in the tissues of naive (uninfected [UN]) and P. yoelii NL (PyNL)-infected recipient mice. (C) The numbers of adoptively transferred CD4^+^ YFP^+^ GFP^−^ T cells (left) and CD4^+^ YFP^+^ GFP^+^ T cells (right) recovered in the tissues of naive and infected recipients. (D, E) IFN-γ and IL-10 dual-reporter mice were infected (i.v.) with 1 × 10^4^ P. yoelii NL pRBCs. (D) Representative histograms showing the expression of chemokine receptors on splenic CD4^+^ YFP^+^ GFP^−^ and CD4^+^ YFP^+^ GFP^+^ T cells on day 7 of infection. (E) Frequencies of CD4^+^ YFP^+^ GFP^−^ and CD4^+^ YFP^+^ GFP^+^ T cells expressing chemokine receptors on day 7 of infection. The results are the mean ± SEM for the group with 3 to 5 mice per group. The results are representative of those from 2 independent experiments. *, *P* < 0.05. Significance was tested using an unpaired *t* test.

The data presented above indicate that CD4^+^ IFN-γ^+^ IL-10^+^ T cells distribute systemically during malaria infection to appropriately balance pro- and anti-inflammatory immune responses. However, whether they migrated to the nonlymphoid tissue sites from the lymphoid compartment as mature and stable cells or whether they formed in nonlymphoid tissues following *in situ* reprogramming of effector CD4^+^ T cells was unknown. To address these questions, we adoptively transferred antigen-experienced splenic YFP^+^ GFP^−^ and YFP^+^ GFP^+^ CD4^+^ T cells, obtained on day 7 of infection, into naive congenic (CD45.1^+^) mice or congenic mice on day 7 of infection. Donor (CD45.2^+^) YFP^+^ GFP^−^ and YFP^+^ GFP^+^ cells were found in the spleen, liver, lung, and blood in both naive and infected recipients at 1 day posttransfer (Fig. 5A and B). The transferred CD4^+^ YFP^+^ GFP^−^ T cells remained predominantly GFP^−^ in all tissues of uninfected and infected recipient mice examined (Fig. 5A and B). In contrast, the CD4^+^ YFP^+^ GFP^+^ T cells stably maintained GFP expression in all examined tissues following transfer (Fig. 5A and B). Interestingly, significantly increased numbers of CD4^+^ YFP^+^ GFP^+^ T cells were recovered in the liver and lungs of infected animals compared with the numbers recovered in the liver and lungs of naive recipients. In contrast, increased numbers of CD4^+^ YFP^+^ GFP^−^ T cells were recovered only in the liver of infected animals compared with the numbers recovered in the liver of naive recipients (Fig. 5C). Combined, these data indicate that upon egress from the spleen, CD4^+^ YFP^+^ GFP^+^ T cells can seed the nonlymphoid organs during malaria infection and that rapid *in situ* reprogramming of CD4^+^ YFP^+^ GFP^−^ T cells does not appear to occur within nonlymphoid tissues during infection. Moreover, CD4^+^ YFP^+^ GFP^+^ T cells are significantly more able to accumulate in inflamed than noninflamed nonlymphoid organs.

To address the molecular mechanism through which splenic antigen-experienced CD4^+^ YFP^+^ GFP^+^ T cells are able to migrate to and enter inflamed organs during malaria infection, we assessed the repertoire of chemokine receptors expressed on their cellular surface. Splenic CD4^+^ YFP^+^ GFP^+^ T cells from mice on day 7 of infection were almost universally CXCR3^+^, whereas they expressed only low levels of CCR1, CCR6, and CXCR6 (Fig. 5D and E). Interestingly, while the expression of these 4 chemokine receptors was not significantly different on CD4^+^ YFP^+^ GFP^+^ and CD4^+^ YFP^+^ GFP^−^ T cells, splenic CD4^+^ YFP^+^ GFP^+^ T cells expressed significantly higher levels of CCR5 than CD4^+^ YFP^+^ GFP^−^ T cells (Fig. 5D and E). Thus, splenic CD4^+^ YFP^+^ GFP^−^ and CD4^+^ YFP^+^ GFP^+^ cells appear to utilize overlapping but also disparate sets of chemokine receptors to migrate during malaria infection.

### CD4^+^ YFP^+^ GFP^+^ T cells exhibit comparable phenotypic profiles but graded functional signatures in lymphoid and nonlymphoid tissues during malaria infection.

**FIG 6:**
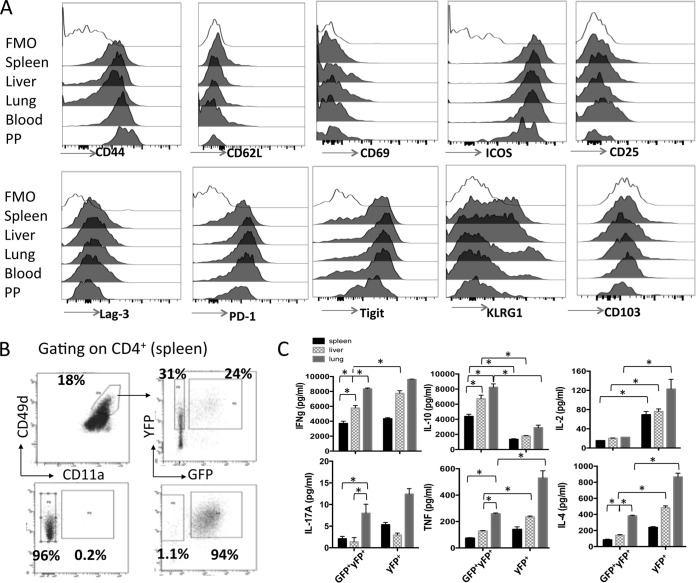
CD4^+^ IFN-γ–YFP^+^ IL-10–GFP^+^ T cells exhibit a conserved phenotype in lymphoid and nonlymphoid organs but display tissue-dependent functional responses during malaria infection. IFN-γ and IL-10 dual-reporter mice were infected (i.v.) with 1 × 10^4^ P. yoelii NL pRBCs. (A) Representative histograms showing the expression of activation and regulation-associated molecules on CD4^+^ IFN-γ–YFP^+^ IL-10–GFP^+^ T cells in the different tissues on day 7 of infection. The results are the mean ± SEM for the group with 3 to 5 mice per group. The results are representative of those from 3 independent experiments. (B, C) Antigen-experienced (CD11a^+^ CD49d^+^) IFN-γ–YFP^+^ GFP^+/−^ CD4^+^ T cells were sort purified from the spleens, liver, and lung of P. yoelii NL-infected (day 7, 1 × 10^4^ pRBCs [i.v.]) dual-reporter mice. (B) Representative dot plots showing the purity of sorted (splenic) CD4^+^ IFN-γ–YFP^+^ GFP^−^ and CD4^+^ IFN-γ–YFP^+^ GFP^+^ T cells. (C) The sorted populations of tissue-derived cells were stimulated *in vitro* in triplicate with anti-CD3 and anti-CD28 for 24 h, and cytokine production in the culture supernatant was assessed using cytokine bead arrays. The results are the mean ± SEM for the group. The results are representative of those from 2 independent experiments. *, *P* < 0.05. Significance was tested using one-way ANOVA with Tukey *post hoc* analysis.

It is becoming clear that Foxp3^+^ regulatory T cells (Tregs) show phenotypic and functional disparities in different anatomical locations during inflammation which are necessary for their compartment-specific regulatory capacity (33, 34). Consequently, we next examined whether the CD4^+^ YFP^+^ GFP^+^ T cells undergo site-specific adaptations during malaria infection that may affect or tune their regulatory capacities in the different tissue environments. Surprisingly, CD4^+^ YFP^+^ GFP^+^ T cells from the spleen, liver, lung, blood, and Peyer's patches of mice on day 7 of infection displayed very few phenotypic differences; all populations expressed high levels of CD44, ICOS, PD-1, and Tigit; intermediate levels of KLRG-1 and Lag-3; and low levels of CD62L, CD69, CD25, and CD103 (Fig. 6A). The CD4^+^ YFP^+^ GFP^+^ T cells exhibited a largely equivalent profile on day 14 of infection (results not shown). Thus, antigen-experienced CD4^+^ YFP^+^ GFP^+^ T cells appear to exhibit a conserved phenotype in both lymphoid and nonlymphoid organs during malaria infection, suggesting that they may not require significant adaptations to regulate effectively within disparate tissue environments.

Although the CD4^+^ YFP^+^ GFP^+^ T cells from the different tissues were phenotypically comparable, we questioned whether they were functionally equivalent on a cell-per-cell basis. CD4^+^ YFP^+^ GFP^+^, CD4^+^ YFP^+^ GFP^−^, and CD4^+^ YFP^−^ GFP^−^ T cells were sort purified from the spleen, liver, and lungs of mice on day 7 of infection (Fig. 6B), and equal numbers of tissue-derived cells were stimulated *in vitro* for 24 h with anti-CD3 and anti-CD28, after which the supernatant was assayed for cytokine production. CD4^+^ YFP^+^ GFP^+^ and CD4^+^ YFP^+^ GFP^−^ T cells from all three tissues produced large amounts of IFN-γ. Although the magnitude of production by cells from the three different tissues varied (the highest level of production was by lung-derived cells, and the lowest was by splenic cells), the levels of production in cultures of CD4^+^ YFP^+^ GFP^+^ and CD4^+^ YFP^+^ GFP^−^ T cells from within each tissue were largely equivalent, being marginally different only in the liver (Fig. 6C). CD4^+^ YFP^+^ GFP^+^ T cells produced significantly more IL-10 than CD4^+^ YFP^+^ GFP^−^ T cells following stimulation, and consistent with the IFN-γ results, the level of IL-10 production was the highest by lung-derived CD4^+^ YFP^+^ GFP^+^ cells and the lowest by splenic-derived CD4^+^ YFP^+^ GFP^+^ T cells (Fig. 6C). Neither IFN-γ nor IL-10 was produced at high levels by CD4^+^ YFP^−^ GFP^−^ T cells (results not shown but provided for review), validating the utility of YFP and GFP in reporting the levels of production of the respective cytokines. Interestingly, CD4^+^ YFP^+^ GFP^+^ T cells from all tissues expressed smaller amounts of IL-2 than the corresponding CD4^+^ YFP^+^ GFP^−^ T cells, whereas CD4^+^ YFP^+^ GFP^+^ T cells from the liver and lung produced less TNF and IL-4 than their tissue CD4^+^ YFP^+^ GFP^−^ T cell counterparts (Fig. 6C). IL-17A was produced at extremely low levels by CD4^+^ YFP^+^ GFP^+^ and CD4^+^ YFP^+^ GFP^−^ T cells from all tissues (Fig. 6C). Consistent with the lack of a Th17 response during malaria infection (35, 36), splenic CD4^+^ YFP^−^ GFP^−^ T cells also failed to produce significant quantities of IL-17A (results not shown but provided for review). Thus, these data suggest that CD4^+^ YFP^+^ GFP^+^ T cells produce graded IL-10 expression in different locations during malaria infection. Moreover, although IL-10 may modify IL-2 production and have tissue-specific effects on IL-4 and TNF production, it does not appear to act in an autocrine manner to suppress IFN-γ production by effector CD4^+^ T cells.

### IL-27 and ICOS control the development and maintenance of CD4^+^ YFP^+^ GFP^+^ T cells in both lymphoid and nonlymphoid compartments during malaria infection.

**FIG 7:**
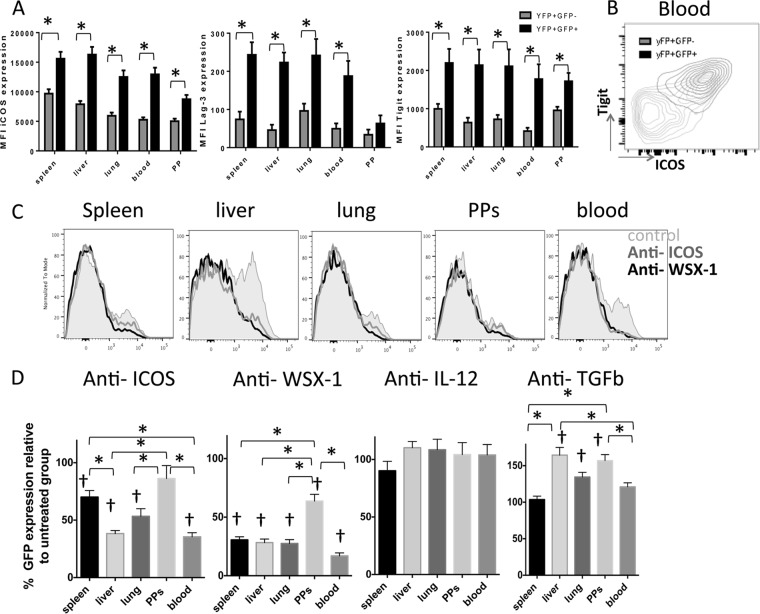
IL-27R and ICOS control IL-10 expression by antigen-experienced CD4^+^ T cells in both lymphoid and nonlymphoid tissues during malaria infection. IFN-γ and IL-10 dual-reporter mice were infected (i.v.) with 1 × 10^4^ P. yoelii NL pRBCs. (A) The mean fluorescence intensity (MFI) of activation- and regulation-associated molecules on antigen-experienced CD4^+^ IFN-γ–YFP^+^ IL-10–GFP^+^ and CD4^+^ IFN-γ–YFP^+^ IL-10–GFP^−^ T cells in the different tissues on day 7 of infection. (B) Representative dot plot showing the coordinated expression of Tigit and ICOS on splenic CD4^+^ IFN-γ–YFP^+^ IL-10–GFP^+^ T cells on day 7 of infection. (C, D) IFN-γ and IL-10 dual-reporter mice were treated with anti-ICOS, anti-WSX-1, anti-IL-12, or anti-TGF-β on days −1, 1, 3, and 5 of P. yoelii NL infection. (C) Representative histograms showing the expression of IL-10–GFP by parasite-specific CD4^+^ IFN-γ–YFP^+^ T cells in the different tissues on day 7 of infection following anti-ICOS or anti-WSX-1 MAb administration. (D) The frequencies of parasite-specific CD4^+^ T cells expressing IL-10–GFP in each tissue on day 7 of infection (relative to control treated group normalized to 100%). The results are the mean ± SEM for the group from 3 to 4 independent experiments (3 to 5 mice in each experiment). *, *P* < 0.05 compared with the control treated group; †, *P* < 0.05 compared with the control treated group. Significance was tested using an unpaired *t* test (A) and one-way ANOVA with Tukey *post hoc* analysis (D).

Finally, we examined the molecular pathways responsible for instruction and/or maintenance of IL-10 expression by parasite-specific CD4^+^ IFN-γ^+^ T cells in the different lymphoid and nonlymphoid compartments during infection. To do this, we first contrasted the expression of various molecules on CD4^+^ YFP^+^ GFP^−^ and CD4^+^ YFP^+^ GFP^+^ T cells that have previously been implicated in driving IL-10 expression (1, 29). Irrespective of tissue origin, ICOS was expressed at significantly higher levels on infection-induced CD4^+^ YFP^+^ GFP^+^ T cells than on the corresponding CD4^+^ YFP^+^ GFP^−^ T cells (Fig. 7A). Furthermore, Lag-3 and Tigit were also preferentially expressed by CD4^+^ YFP^+^ GFP^+^ T cells (albeit at relatively low levels in the case of Lag-3; Fig. 7A), with ICOS and Tigit being coordinately expressed (Fig. 7B). There were no significant differences in the levels of expression of CD103, CD69, PD-1, CD25, or KLRG-1 by CD4^+^ YFP^+^ GFP^−^ and CD4^+^ YFP^+^ GFP^+^ T cells in any examined location (results not shown).

Given the differential expression of ICOS by CD4^+^ YFP^+^ GFP^−^ and CD4^+^ YFP^+^ GFP^+^ T cells and its documented role in promoting IL-10 production by CD4^+^ T cells in other models (37, 38), we directly assessed the importance of ICOS in promoting IL-10 expression by CD4^+^ YFP^+^ T cells in the various lymphoid and nonlymphoid locations during malaria infection. Moreover, we assessed whether IL-27 controls IL-10 production by CD4^+^ T cells only in the spleen during infection (22) or whether it exerts a dominant role in shaping the nonlymphoid CD4^+^ IL-10^+^ T cell response. Blockade of ICOS and IL-27R by administration of antibodies from day −1 of infection did not substantially alter the frequencies of total antigen-experienced (CD11a^+^ CD49d^+^) CD4^+^ or CD4^+^ YFP^+^ T cells in any examined organ, indicating that neither ICOS nor IL-27R is important for development of the proinflammatory parasite-specific CD4^+^ T cell response during infection (results not shown). Furthermore, anti-ICOS and anti-WSX-1 administration did not significantly alter the level of peripheral parasitemia (up to day 7 of infection, when the experiment was terminated). Importantly, ICOS blockade significantly reduced the frequencies and number of CD4^+^ YFP^+^ GFP^+^ T cells in the spleen, liver, lung, and blood but not the Peyer's patches compared with those in control treated mice (Fig. 7C and D). The effect of anti-ICOS blockade was, however, heterogeneous and tissue dependent, with the strongest suppression of IL-10 expression by parasite-specific CD4^+^ T cells being observed in the liver and blood compared with its level of expression in the spleen (Fig. 7C and D). In contrast to the apparent tissue-specific effect of anti-ICOS administration, blockade of IL-27R activity significantly and uniformly reduced the frequencies of IL-10-expressing CD4^+^ T cells by >70% in all examined tissue sites, with the exception of the Peyer's patches (Fig. 7C and D). Blockade of TGF-β resulted in increased production of IL-10 by CD4^+^ T cells in the liver, lung, and Peyer's patches (Fig. 7D), suggesting that during malaria infection TGF-β inhibits IL-10 production by effector CD4^+^ YFP^+^ T cells, possibly by repressing the strength of cell activation (29). Neutralization of IL-12p40 and IL-2 and blockade of CD70 failed to modify IL-10 expression by CD4^+^ T cells in any examined location during infection, indicating that these pathways do not control IL-10 production during malaria infection (Fig. 7D and results not shown). Due to the current unavailability of antagonistic anti-Tigit antibodies, we were unable to directly assess the importance of Tigit in promoting IL-10 expression by CD4^+^ T cells during malaria infection.

**FIG 8:**
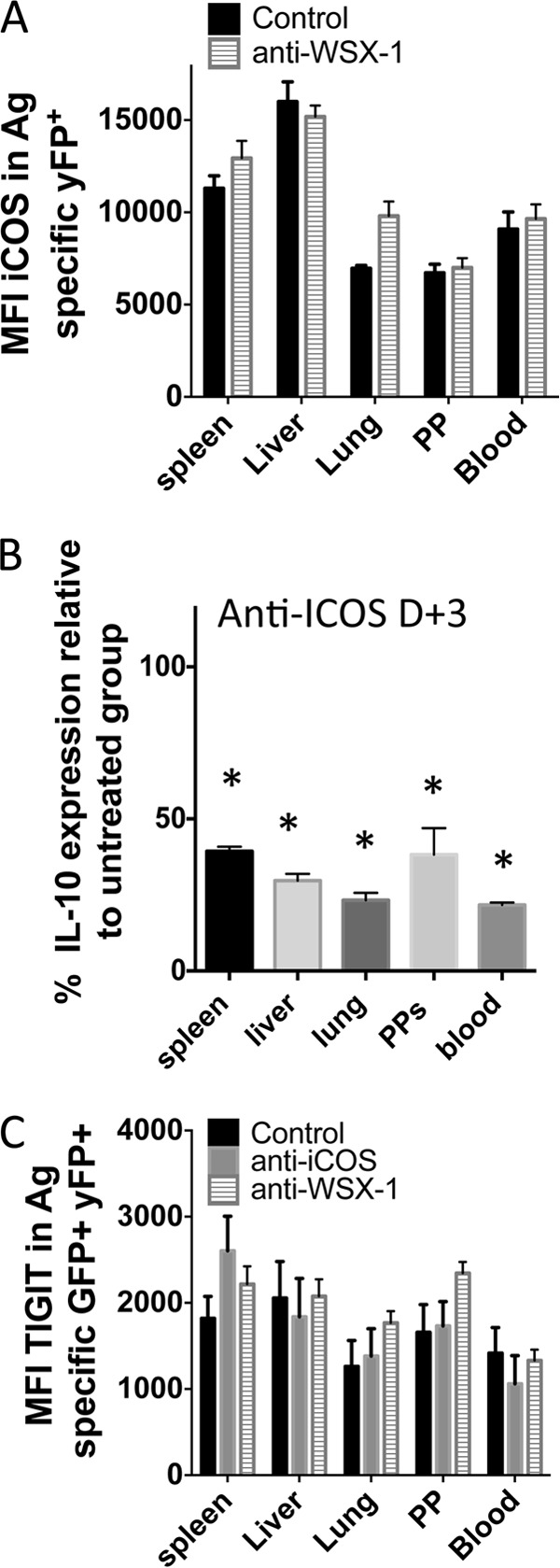
ICOS signaling after T cell priming actively promotes IL-10 expression by antigen-experienced CD4^+^ T cells during malaria infection. IFN-γ and IL-10 dual-reporter mice were infected (i.v.) with 1 × 10^4^ P. yoelii NL pRBCs. (A) The mean fluorescence intensity (MFI) of ICOS expression on parasite-specific CD4^+^ IFN-γ–YFP^+^ T cells in the different tissues of mice treated with control or anti-WSX-1 MAbs (MAb was administered on days −1, 1, 3, and 5 of infection, analysis was on day 7 of infection). (B) The relative frequencies of parasite-specific CD4^+^ T cells expressing IL-10–GFP in the tissues of mice treated with control or anti-ICOS MAbs (MAb was administered on day 3 [D+3] and day 5 of infection, analysis was on day 7 of infection). (C) The MFI of Tigit expression on parasite-specific CD4^+^ IFN-γ–YFP^+^ GFP^+^ T cells in the different tissues of mice treated with control, anti-WSX-1, or anti-ICOS MAbs (MAb was administered on days −1, 1, 3, and 5 of infection, analysis was on day 7 of infection). The results are the mean ± SEM for the group with 3 to 5 mice per group. The results are representative of those from 2 independent experiments. *, *P* < 0.05 compared with the control treated group. Significance was tested using an unpaired *t* test (A) and one-way ANOVA with Tukey *post hoc* analysis (B, C).

We observed that blockade of IL-27R did not significantly modify the expression of ICOS on parasite-specific CD4^+^ YFP^+^ T cells (Fig. 8A). Thus, ICOS expression by itself appeared to be insufficient to promote IL-10 production by CD4^+^ YFP^+^ T cells in the absence of IL-27R signaling. We consequently questioned whether ICOS plays a role in the subsequent stabilization or maintenance of IL-10 expression by CD4^+^ T cells during infection potentially downstream of the IL-27R-dependent induction of IL-10 expression. In support of this hypothesis, blockade of ICOS from day 3 of infection, which is after CD4^+^ T cell priming (39), significantly abrogated the expression of IL-10 by parasite-specific CD4^+^ T cells in all examined organs (Fig. 8B). Interestingly, inhibition of IL-10 expression appeared to be more pronounced, particularly in the spleen and lung, when treatment was commenced on day 3 of infection than when it was commenced treatment from day −1 (Fig. 7C and D). Nevertheless, blockade of neither ICOS nor IL-27R fully attenuated IL-10 expression by CD4^+^ T cells, indicating that other IL-10-inducing pathways may operate during malaria infection. Tigit expression by antigen-experienced CD4^+^ GFP^+^ YFP^+^ T cells was not altered following blockade of IL-27R or ICOS signaling (Fig. 8C). This suggests that the Tigit pathway could work independently from ICOS and IL-27R to drive low-level IL-10 expression by CD4^+^ T cells during blood-stage malaria infection. A role for Tigit in promoting IL-10 production by CD4^+^ T cells has been reported (40, 41). Taken together, these results demonstrate the importance of IL-27R, ICOS, and, potentially, Tigit signaling in promoting and maintaining IL-10 production by CD4^+^ T cells in lymphoid and nonlymphoid compartments during malaria infection.

## DISCUSSION

In this study, we have shown, using novel IFN-γ–YFP and IL-10–GFP dual-reporter mice, that antigen-experienced and not homeostatic or bystander-activated effector CD4^+^ IFN-γ^+^ T cells are the dominant source of IL-10 in both lymphoid and nonlymphoid compartments throughout the course of malaria infection. Thus, although CD4^+^ T cells (Th1 cells) have previously been shown to be the primary source of IL-10 in the spleen during murine malaria infection (22) and in the blood during human malaria infection (24, 25), this is the first study to definitively address the cellular source of this nonredundant and critical cytokine in nonlymphoid organs, including the liver and lung, where pathology occurs in IL-10-deficient mice during malaria infection. Interestingly, the percentage of CD4^+^ YFP^+^ T cells that coproduced GFP reached an equivalent upper plateau in all examined organs (with the exception of the Peyer's patches), suggesting that there may be an upper threshold or requirement of IL-10 production by the parasite-specific CD4^+^ YFP^+^ T cell population in discrete tissue sites during malaria infection. Similar frequencies of antigen-experienced CD4^+^ GFP^+^ T cells were observed during malaria infection in homozygous Vert-X mice and in heterozygous YETI-Vert-X mice, indicating that the threshold of IL-10 expression was not due to the monoalleleic expression of the IL-10 gene (results not shown).

Our results provide important information on the developmental relationships between parasite-specific CD4^+^ YFP^+^ GFP^+^ T cells found in the lymphoid and nonlymphoid compartments during malaria infection. We found that adoptively transferred mature splenic CD4^+^ YFP^+^ GFP^+^ T cells from malaria parasite-infected mice were able to migrate to and accumulate within the nonlymphoid organs of malaria parasite-infected mice and, to a much lesser extent, naive mice. Critically, we also found that while adoptively transferred splenic CD4^+^ YFP^+^ GFP^−^ T cells were also able to migrate to and accumulate in nonlymphoid organs of naive and malaria parasite-infected mice, they did not appear to rapidly upregulate IL-10 expression upon entry into the nonlymphoid tissues. The latter result was unexpected, as it has previously been shown in other models of inflammation that cells residing in the liver and lung can repress activated T cell effector functions and promote Treg development through mechanisms including PD-L1, TGF-β, and CD200 (26, 27, 42). Moreover, the liver can also acquire lymphoid tissue characteristics during inflammation, with resident antigen-presenting cells orchestrating the priming of naive T cells (43). Thus, while we cannot entirely discount the possibility that some endogenous migrating CD4^+^ YFP^+^ GFP^−^ cells can gradually be reprogrammed to express IL-10 *in situ* within the inflamed tissues during malaria infection, our data support a model where IL-10 expression by CD4^+^ T cells is instructed principally in the lymphoid compartment during malaria infection. This could occur following conversion of IFN-γ^+^ single producing effector CD4^+^ T cells or through direct priming of naive cells to the IFN-γ^+^ IL-10^+^ phenotype (29, 44). Although not definitively shown, these CD4^+^ IFN-γ^+^ IL-10^+^ T cells appear to be capable of transiting via the blood to enter and accumulate in inflamed nonlymphoid tissues to dampen tissue-damaging inflammation.

The CD4^+^ YFP^+^ GFP^+^ T cells expressed equivalent amounts of CXCR3 but larger amounts of CCR5 compared with the amounts expressed by CD4^+^ YFP^+^ GFP^−^ T cells. Thus, inflammatory and regulatory CD4^+^ T cell subsets appear to be able to utilize an equivalent CXCR3-dependent mechanism to migrate during malaria infection. However, an altered responsiveness to CCR5 ligands may provide a mechanism for preferential recruitment of the regulatory rather than proinflammatory cells to dampen tissue inflammation. Notably, CCL4 and CCL5 are produced by inflamed tissues during malaria infection (45, 46).

Surprisingly, we did not observe any obvious or extensive differences in the phenotypic profiles of CD4^+^ YFP^+^ GFP^+^ cells obtained from the different tissue sites during infection. While we cannot discount the possibility that CD4^+^ YFP^+^ GFP^+^ T cells from different tissues differentially express other molecules that were not examined in his study, our results suggest that, as opposed to Foxp3^+^ Tregs (33, 34), the CD4^+^ YFP^+^ GFP^+^ T cells may not undergo the wide-scale tissue-dependent adaptations necessary for optimization of the localized regulatory function in discrete tissue sites. Nevertheless, parasite-specific CD4^+^ YFP^+^ GFP^+^ T cells in the liver and lung exhibited graded increases in IL-10 production compared with cells from the spleen. Consequently, it is possible that the functional activity of the cells is variably instructed upon entry into distinct anatomical locations. Alternatively, as CD4^+^ YFP^+^ GFP^+^ cells in the liver and lung produced higher levels of IFN-γ than cells in the spleen, it is possible that the level of IL-10 production is directly correlated with and determined by the amount of IFN-γ produced.

Our results have also provided information on the signals that control IL-10 expression by CD4^+^ IFN-γ^+^ T cell populations systemically during malaria infection. We found not only that IL-27 instructs IL-10 expression by CD4^+^ IFN-γ^+^ T cells within the spleen during malaria infection, as previously shown (22), but also that it controls IL-10 production by CD4^+^ T cells in nonlymphoid tissues during infection. However, as importantly, we found that ICOS also plays a role in controlling IL-10 production by CD4^+^ YFP^+^ T cells during infection. Neither IL-27 nor ICOS played a role in the development of parasite-specific CD4^+^ YFP^+^ T cell responses during infection, implying that these pathways specifically modify the induction of IL-10 expression, rather than modifying the initial development of the Th1 response.

Since IL-27 has been shown to promote IL-10 production by Tr1 cells via an ICOS-dependent mechanism (47), it is feasible that ICOS simply acts downstream of IL-27R signaling to control IL-10 production by CD4^+^ IFN-γ^+^ T cells during malaria infection. Thus, it is notable that anti-ICOS administration from day 3 of infection, after initial CD4^+^ T cell priming and differentiation events (39), led to impaired IL-10 production by parasite-specific CD4^+^ YFP^+^ T cells. Consequently, combined, our results support the conclusion that IL-27R signaling is critical for the initial differentiation of CD4^+^ IFN-γ^+^ IL-10^+^ T cells, which appears to occur in the spleen during malaria infection. IL-27 may modify the epigenetic landscape of the IL-10 gene and promote the binding of transcription factors, such as c-Maf, Ahr, or Blimp-1, which are necessary for initial transcription of the IL-10 gene (1). Following differentiation, the CD4^+^ IFN-γ^+^ IL-10^+^ T cells transition into a new state, where context-dependent pathways, such as ICOS, are required to stabilize or maintain IL-10 expression in the different tissue environments. The increased effectiveness of anti-ICOS administration in inhibiting IL-10–GFP expression from day 3 of infection compared with its effectiveness with administration from day −1 is potentially due to the development of compensatory immune mechanisms when administration was commenced before immune priming, which subsequently reduced the relative importance of ICOS in maintaining IL-10 expression in CD4^+^ YFP^+^ T cells. Further work is required to resolve the networks of transcription factors and immune signals that work in a temporally and spatially coordinated fashion to control and subsequently maintain IL-10 expression in CD4^+^ T cells and to decipher the number of context-dependent pathways that can independently promote IL-10 responses during inflammation and infection.

Although the results of this study significantly enhance our understanding of the cellular sources of host-protective IL-10 during malaria infection and the signals required for instructing and/or maintaining expression in distinct lymphoid and nonlymphoid tissue sites, the mechanisms through which IL-10 limits immune-mediated pathology during infection remain unclear. Our results show that, irrespective of tissue origin, CD4^+^ YFP^+^ GFP^+^ cells produced IFN-γ at levels comparable to those for their counterpart CD4^+^ YFP^+^ GFP^−^ T cells. Thus, IL-10 may not work in an autocrine loop targeting producing CD4^+^ T cells during malaria infection. Of relevance, we have also previously observed that, in contrast to the results obtained with IL-27R-deficient mice, Th1 responses in IL-10- and IL-10 receptor (IL-10R)-deficient mice are largely comparable to those obtained with wild-type mice during malaria infection (48, 49). Consistent with observations made during Toxoplasma gondii infection (44), these data argue that IL-10 may act principally by suppressing non-CD4^+^ T cell responses, such as cells of the innate system, during malaria infection to prevent immune-mediated pathology.

In summary, by performing a detailed spatiotemporal examination of the IL-10 response during malaria infection, we have significantly expanded our knowledge of the sources of this critical cytokine. We have resolved how CD4^+^ IFN-γ^+^ IL-10^+^ T cells distribute systemically within nonlymphoid tissues during infection to limit inflammation, and we have identified pathways that orchestrate IL-10 production by effector CD4^+^ IFN-γ^+^ T cells, potentially at distinct phases of infection. These investigations provide insights that may subsequently lead to the identification of mechanisms to therapeutically tune activated T cell responses to modify the strength and nature of organ-specific immune responses.
